# Public health round-up 

**DOI:** 10.2471/BLT.17.011117

**Published:** 2017-11-01

**Rohingya and host communities receive cholera vaccine**

A massive cholera immunization campaign was launched on 10 October near Cox’s Bazar in Bangladesh to protect newly arrived Rohingyas and their host communities. About 900 000 doses of the vaccine are being delivered by 200 vaccination teams. The campaign led by the health ministry is supported by the World Health Organization (WHO) and other partners.

http://www.searo.who.int/mediacentre/emergencies/bangladesh-myanmar

**Figure:**
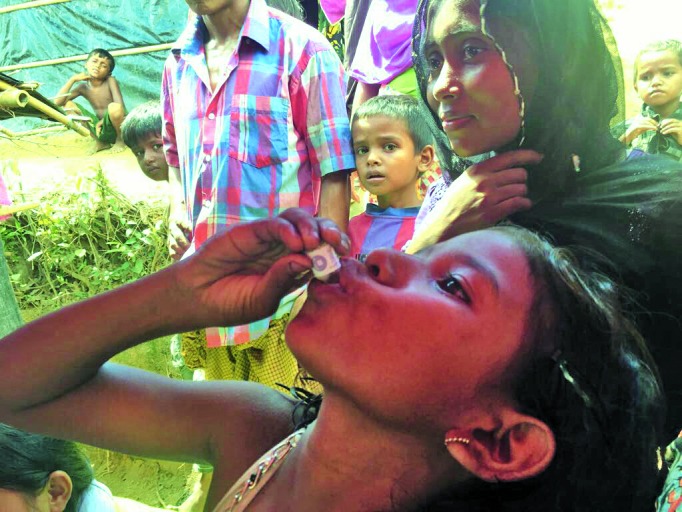
WHO SEARO/M. Vurens van Es

## New WHO commission on noncommunicable diseases

The World Health Organization (WHO) is setting up a High-level Global Commission on Noncommunicable Diseases (NCDs) to find innovative ways to fight the world’s biggest causes of death.

The commission will be chaired by Dr Sania Nishtar, a prominent global advocate for action against NCDs who co-chaired the WHO Commission on Ending Childhood Obesity from 2015 to 2016.

The commission will support efforts in countries to address cardiovascular disease, cancers, diabetes and respiratory disease as well as mental health issues and injuries.

In 2015, world leaders committed to reducing premature deaths from NCDs by one third by 2030, as part of the sustainable development goals. Recent data suggest that many countries are struggling to meet that target.

“We urgently need new approaches and action on a dramatically different scale if we are to stop people dying unnecessarily from noncommunicable diseases,” said Dr Tedros Adhanom Ghebreyesus, WHO Director-General, who announced the establishment of the commission on 10 October. 

Last month government ministers and other health leaders from around the world reviewed progress countries are making in the fight against NCDs at the WHO Global Conference on Noncommunicable Diseases in Montevideo, Uruguay.

www.who.int/mediacentre/news/statements/2017/ncd-commission

## Child obesity set to surpass child underweight

The number of obese children and adolescents worldwide has increased tenfold over the past four decades according to a study led by Imperial College London and WHO.

If current trends continue, more children and adolescents will be obese than moderately or severely underweight by 2022, according to the study that was published in The Lancet ahead of World Obesity Day on 11 October.

The study presents the first ever comprehensive data on underweight through to obesity for children and adolescents aged five to 19 years old.

To coincide with the launch of these data, WHO published a summary of key recommendations from the implementation plan for the *Report of the commission on ending childhood obesity*. The report provides guidance to countries on the effective actions they can take to curb childhood and adolescent obesity.

Action to curb obesity and malnutrition are key elements of the *United Nations 2030 agenda for sustainable development*.

www.who.int/end-childhood-obesity/news/new-estimate-child-adolescent-obesity

## Zoonotic tuberculosis roadmap

Close collaboration is needed between specialists working on human and animal health to combat bovine tuberculosis in animals and prevent its transmission to humans, known as zoonotic tuberculosis, according to a new WHO roadmap to combat this neglected form of tuberculosis. 

Zoonotic tuberculosis is usually transmitted to humans through the consumption of dairy products or, less commonly, contact with contaminated meat from diseased animals. Airborne transmission from infected animals to humans can also occur.

The *Roadmap for zoonotic tuberculosis* was launched on 12 October at the 48th Union World Conference on Lung Health, which took place in Guadalajara, Mexico. 

It was developed by WHO and its partners, the World Organisation for Animal Health, the Food and Agriculture Organization of the United Nations and the International Union Against Tuberculosis and Lung Disease. 

WHO estimates more than 140 000 new cases and 12 000 deaths every year due to zoonotic tuberculosis. These cases are mostly in Africa and south-east Asia. The impact of the disease extends beyond human health. Bovine tuberculosis threatens animal health and welfare, with a major economic impact on livelihoods and trade.

www.who.int/tb

## Plague in Madagascar

WHO delivered nearly 1.2 million doses of antibiotics and released US$ 1.5 million in emergency funds to fight a plague outbreak in Madagascar last month.

Plague is endemic to Madagascar and some 400 cases of – mostly bubonic – plague are reported annually.

The current outbreak, however, is different – with a much larger proportion of pneumonic cases affecting new areas of Madagascar, including large urban areas, thereby increasing the risk of transmission.

As of 11 October, 500 cases of plague – including 55 deaths – had been identified, higher than the number expected at this time of year.

A WHO team of 20 specialists in many fields including incident management, surveillance and field epidemiology, and clinical care were deployed to the island state to support 25 staff from the WHO country office, who are dedicated to the plague response.

“Plague can be cured when people receive prompt treatment with antibiotics. We are working with the government and partners to ensure that people know this and that adequate supplies of antibiotics are in place,” said Dr Charlotte Ndiaye, WHO Representative in Madagascar.

WHO’s partners in the response include the United Nations Children’s Fund, the International Federation of Red Cross and Red Crescent, the Madagascar Red Cross, Médecins Sans Frontières, Médecins du Monde and technical specialists from the Global Outbreak Alert and Response Network.

Plague bacteria *(Yersinia pestis)* are transmitted to humans by the bite of infected fleas carried by rats and other rodents, while pneumonic plague is transmitted from person to person. The last plague outbreak reported in Madagascar was in 2016.

www.who.int/mediacentre/news/releases/2017/antibiotics-plague-madagascar

**Cover photo**

A 70-year-old woman who fled Myanmar with her daughter and grandchildren and who was living in the Ban Mai Noi Soi refugee camp in northern Thailand in 2013.

**Figure:**
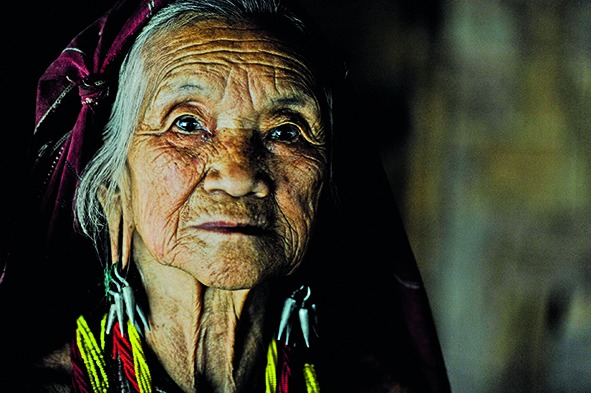
©UNHCR/Sebastian Rich

## Syphilis screening 

All pregnant women should be screened for syphilis at their first antenatal care visit to prevent fetal and newborn complications, according to new WHO recommendations released last month.

Screening of pregnant women for syphilis should be done through “on-site” tests – i.e. tests carried out at the clinic where the antenatal care is provided – in places where coverage of syphilis screening and treatment services is low, where loss to follow-up of pregnant women is high and where laboratory capacity is limited.

A single on-site rapid test should also be used to screen for syphilis in places where prevalence is low – below 5% – and patients should be treated if they test positive.

In places where syphilis prevalence is high, an on-site rapid test followed by prompt treatment for a positive result is also recommended, this should be followed by a rapid plasma reagin test (RPR), and – if that test is positive – provision of treatment.

The new guideline recommends this sequence of tests and treatment rather than the single on-site RPR test previously recommended in high prevalence settings.

The new recommendations are based on recent evidence and available serological tests for syphilis, and they appear in WHO *Guidelines on syphilis screening and treatment for pregnant women*.

www.who.int/reproductivehealth/publications/rtis/syphilis-ANC-screenandtreat-guidelines

## Care for older people

WHO released new guidance for community-based health-care providers on how best to detect and manage the declining physical and mental capacities of people as they grow older.

These standards, released last month, can be used as the basis for national guidelines and to inform the design of health programmes for older people implemented in primary care settings. 

The publication, entitled *Guidelines on community-level interventions to manage declines in intrinsic capacity*, is divided into three parts.

The first covers declines in intrinsic capacity, including mobility loss, malnutrition, visual impairment and hearing loss, cognitive impairment, and depressive symptoms. The second covers geriatric syndromes associated with care dependency, including urinary incontinence and risk of falls. The third is on the interventions needed to support caregivers.

www.who.int/ageing/publications/guidelines-icope

## TB guidelines compendium

WHO released a collection of its recent guidelines and policy advice standards last month covering a wide range of tuberculosis services, from diagnosis, treatment and care to a patient-centred approach to the delivery of tuberculosis care.

The *Compendium of WHO guidelines and associated standards: ensuring optimum delivery of the cascade of care for patients with tuberculosis* comprises 33 tuberculosis standards, thus consolidating current WHO tuberculosis policy recommendations into a single resource.

The compendium can be used to facilitate planning and delivery of tuberculosis services in countries. It will be regularly updated, including in its digital format, to incorporate new evidence as it emerges.

apps.who.int/iris/bitstream/10665/259180/1/9789241512572-eng.pdf

**Looking ahead**

1–3 November – World Hepatitis Summit 2017, São Paulo, Brazil

5–6 November – G7 Health Ministers’ Meeting, Milan, Italy

16–17 November – Global Ministerial Conference on Tuberculosis, Moscow, Russian Federation

13–19 November – World Antibiotic Awareness Week

